# High-performance perovskite solar cell using photonic–plasmonic nanostructure

**DOI:** 10.1038/s41598-020-67741-9

**Published:** 2020-07-09

**Authors:** Alireza Tooghi, Davood Fathi, Mehdi Eskandari

**Affiliations:** 1 Department of Electrical and Computer Engineering, Tarbiat Modares University (TMU), Tehran, Iran; 2 Nanomaterial Research Group, Academic Center for Education, Culture & Research (ACECR) on TMU, Tehran, Iran

**Keywords:** Solar cells, Nanophotonics and plasmonics, Solar cells

## Abstract

In this paper, a coupled optical-electrical modeling method is applied to simulate perovskite solar cells (PSCs) to find ways to improve light absorption by the active layer and ensure that the generated carriers are collected effectively. Initially, a planar structure of the PSC is investigated and its optical losses are determined. To reduce the losses and enhance collection efficiency, a convex light-trapping configuration of PSC is used and the impacts of these nanostructures on all parts of the cell are investigated. In this convex nanostructured PSC, the power conversion efficiency (PCE) is found to be increased when the thickness of the absorbing layer remained unchanged. Then, a plasmonic reflector is applied to trap light inside the perovskite. In this structure, by scattering light through the surface plasmon resonance (SPR) effect of the Au back-contact, the electromagnetic field is found to concentrate in the active layer. This results in increased perovskite absorption and, consequently, a high current density of the cell. In the final structure, which is the integration of these two structures, optical losses are found to be greatly diminished and the short-circuit current density (J_sc_) is increased from 18.63 mA/cm^2^ for the planar structure to 23.5 mA/cm^2^ for the proposed structure. Due to the increased J_sc_ and open-circuit voltage (V_oc_) caused by the improved carrier collection, the PCE increases from 14.62 to 19.54%.

## Introduction

In recent years, mixed organic–inorganic halide perovskite solar cells have been the subject of intense research because of their attractive properties such as high carrier mobility, suitable bandgap, high open-circuit voltage, and long diffusion length^1–4^. In addition, features such as a simple manufacturing process, low fabrication cost, and good efficiency^5–9^ have made these cells competitive to conventional silicon-based crystalline solar cells.

In the development of non-traditional solar cells, the optical and electrical device physics of the components must be considered. Simulation studies are usually performed on these two characteristics separately in order to optimize efficiency. However, simultaneous simulations of both optical and electrical characteristics would afford better understanding of the mechanisms of action. Accordingly, in the present study, a model based on both optical and electrical properties is developed to simulate the nanostructure of perovskite-based solar cells. With such a model, it is possible to simultaneously examine and optimize cell properties such as carrier extraction, recombination, and light absorption.

In thin-film solar cells, all the incident light energy of the spectrum is not fully absorbed by the active layer. Nanostructures are often used to increase the efficiency of energy absorption. Nanostructures enhance light absorption by increasing the pathlength of incident light and trapping it inside the active layer. Nanostructures are also used to facilitate carrier transport to improve electrical properties^10–14^.

Plasmonic and nano-photonic structures have been used to enhance optical absorption^15–19^. In general, in photonic nanostructures, the optical pathlengths inside the absorbing layer and their interaction times can be increased through manipulation of the structure, resulting in enhanced optical absorption. In plasmonic nanostructures, light is efficienly absorbed and trapped through the phenomenon of surface plasmon resonance (SPR) of metal nanoparticles such as silver and gold. In these structures, depending on the size, shape, and distribution of the embedded nanoparticles, two SPR mechanisms—localized near-field modes and far-field scattering effects—are used to either confine the light in the active layer or increase its effective pathlength in this layer^20^. The presence of nanostructures leads to general changes in the absorption mechanism of such structures and induces changes in the intensity and location of the electric field depending on factors such as scattering, localized modes, and guided modes^21^.

In this study, convex nanostructures produced by conformal layer deposition were used to enhance the absorption of the active layer. Such a nanostructure has two advantages: (1) it reduces recombination losses due to high spatial curvature and (2) it can be fabricated easily by processes such as nanoimprint lithography^22,23^. Such a structure has a high potential for both photonic and plasmonic light trapping through redirection of the light path.

In this work, first, a planar solar cell was simulated and analyzed, and introduced as a reference for subsequent investigations. Optical losses in this structure were studied. A structure with an additional anti-reflection layer (ARL) was proposed to reduce reflection from the cell surface. Then, nanostructures with convex portions at different interfaces were designed, to study the efficiency of trapping light in the absorbing layer. The effects of these nanostructures on all parts of the cell were studied and the mechanisms of light-trapping in this configuration were investigated. Subsequently, a plasmonic nanostructure was used to retrieve the unabsorbed light into the perovskite and confine it to this layer. The type of the SPR mechanism that occurs in the plasmonic nanostructure was investigated in terms of the electric field profile. The effects of plasmonic scattering on the light-trapping mechanism of the planar and photonic structures were studied and the light absorption behaviors in these structures were compared. Finally, the electrical properties of the proposed structures were investigated and the best structure was chosen based on the cell efficiency.

**Fig. 1:**
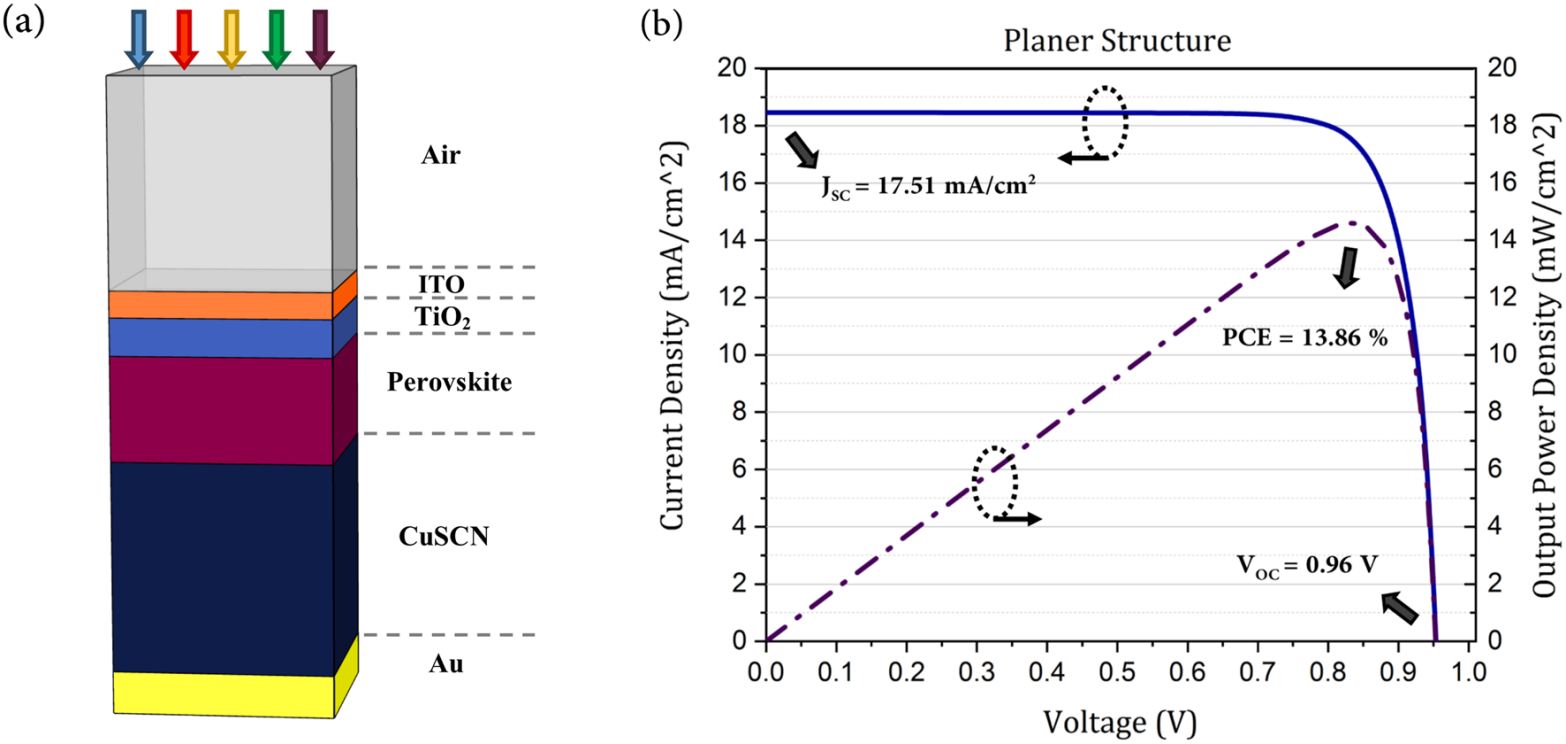
Illustration and characteristic of the conventional planar PSC. (**a**) The schematic of the planar structure of a PSC. (**b**) The current–voltage (J–V) characteristic of a PSC.

**Fig. 2:**
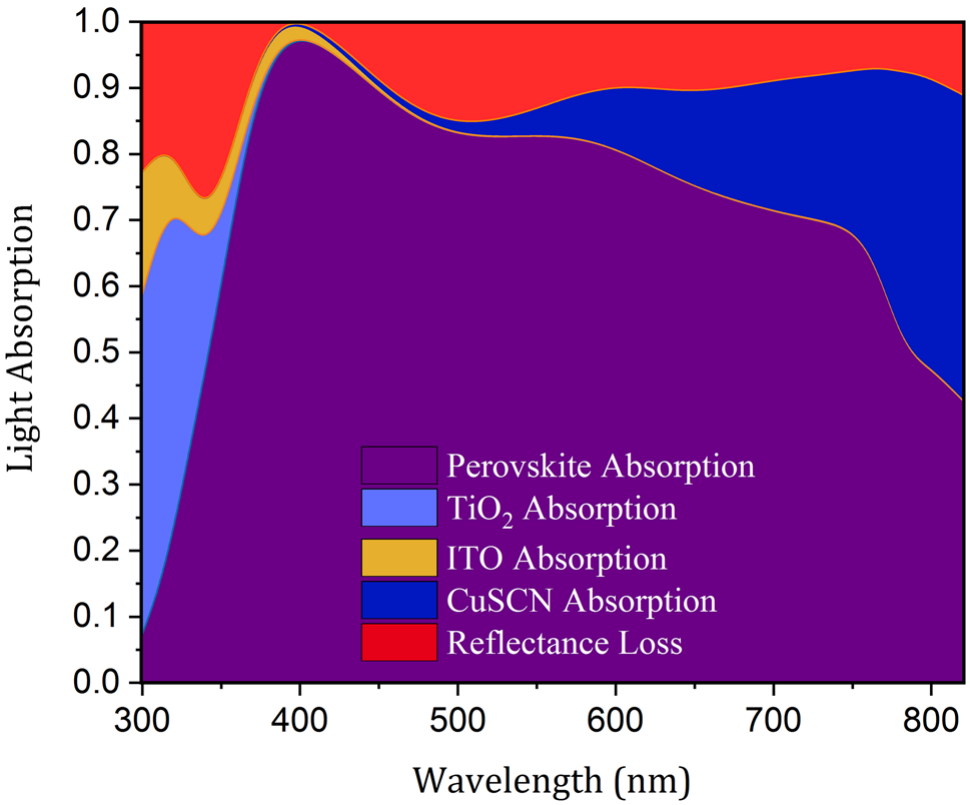
Spectral normalized absorption of each layer of the planar structure of a PSC.

## Results

The planar structure of a PSC was simulated in order to validate the numerical model and to use it as the reference structure for our subsequent simulations. As presented in Fig. 1a, this structure included five layers of Indium Tin Oxide (ITO) as front contact, compact titanium dioxide (TiO_2_) as electron transport layer (ETL), perovskite as absorbing layer, copper thiocyanate (CuSCN) as hole transport layer (HTL), and Au as back-contact. TiO_2_ is a common electron transporting material (ETM) in PSCs due to its suitable energy band levels and ease of nanoparticle fabrication. Generally, in PSCs, the ETM is introduced as a single compact film or in conjunction with a porous film of differently sized nanoparticles to improve carrier extraction. However, highly compact layers of TiO_2_, which can be fabricated easily by such processes as ultrasonic spray pyrolysis (USP) of precursor, atomic layer deposition (ALD), etc., have been shown to improve the cell performance by preventing interfacial carrier recombination and increasing shunt resistivity^24,25^. The highly dense pinhole-free layer of TiO_2_ acts as a hole blocking layer and improves the efficiency of the solar cell, in addition to being a carrier extraction channel^24^. The data of ALD-TiO_2_-based on anatase crystal size between 8 and 23 nm was used in this study^26,27^.

As mentioned earlier, the HTL of this structure was CuSCN, which is a cheap and effective inorganic hole transporting material (HTM) that was first used at PSC in 2014^28^. CuSCN possesses good properties such as large bandgap, high hole mobility, and cost-effective fabrication process. But its most important feature is the match of energy levels of its conduction band to that of the perovskite, which leads to reduced trap-state density, improved carrier extraction, and reduced carrier recombination^28^. It has been shown that the improved quality of the interfacial contact between the hole extraction layer of CuSCN and perovskite and the resulting improvements in carrier extraction result in reduction in the hysteresis behavior of the cell. This increases the filling factor (FF) of the PSC due to suppression of recombination and ultimately improves cell efficiency^29,30^. In our simulations, the thicknesses of the PSC layers were taken as 50, 90, 200, 600, and 100 nm. Figure 1b presents the plot of current density and output power in terms of voltage for this planar structure. J_sc_, V_oc_, FF, and PCE for this structure were 18.63 mA/cm^2^, 0.9564 V, 82.84%, and 14.62%, respectively. These results are consistent with the observations in earlier experimental and numerical studies^28,31^.

Figure 2 presents the absorption of each layer and the total reflection spectrum of the planar structure. This figure shows the amount of incident light energy reflected and absorbed at each layer at each wavelength. By calculating the ratio of the absorbed light in the perovskite to the total incident light over the computational wavelength range, it is seen that only 69% of the incident light was absorbed in the active layer. It can be seen that there are two types of optical losses in the structure. The first is the reflection loss, which means a part of the light does not enter the structure at all. The second type is parasitic losses. As shown in the spectrum, layers other than the active layer absorbed light without concomitant generation of current. The upper layers of the cell were found to absorb blue light, which is of short wavelength ($\lambda<$ 550 nm) and thus, higher energy. The lower layers did not interact with blue light because this part of the light spectrum was fully absorbed before reaching the lower layers, owing to its higher energy. In contrast, the red light, i.e., the longer wavelengths of the light spectrum (550 nm > $\lambda$) with lower energy, was not absorbed in the upper layers. Thus, a part of it that entered the cell was absorbed by the perovskite and the rest penetrated the CuSCN layer. Therefore, the absorption of this layer was at wavelengths above 550 nm. The present study aims to provide a structure to reduce these losses and increase active layer absorption.

**Fig. 3:**
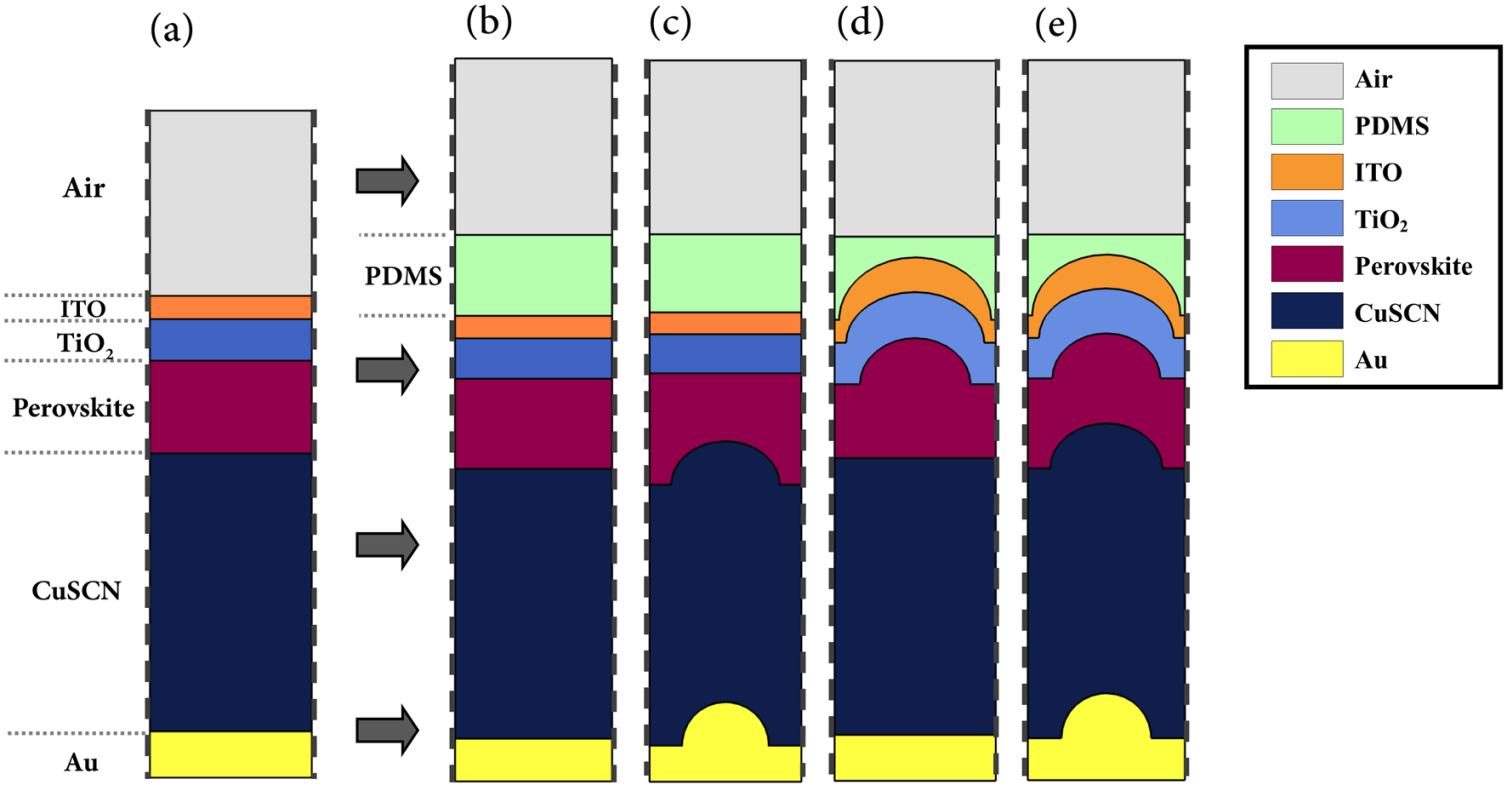
Schematic of the planar and proposed light-trapping structures. The width of the simulated unit cell is 350 nm. The curved Au in (c,e) is a semi-circle with a radius of 100 nm. All other convex nanostructures are semi-ellipses. The curved HTL in (c,e) has a minor radius of 100 nm and a major radius of 110 nm, the curved perovskite has a minor radius of 100 nm and a major radius of 120 nm, the curved ETL has a minor radius of 110 nm and major radius of 150 nm, and the curved ITO has a minor radius of 135 nm and major radius of 165 nm.

**Fig. 4:**
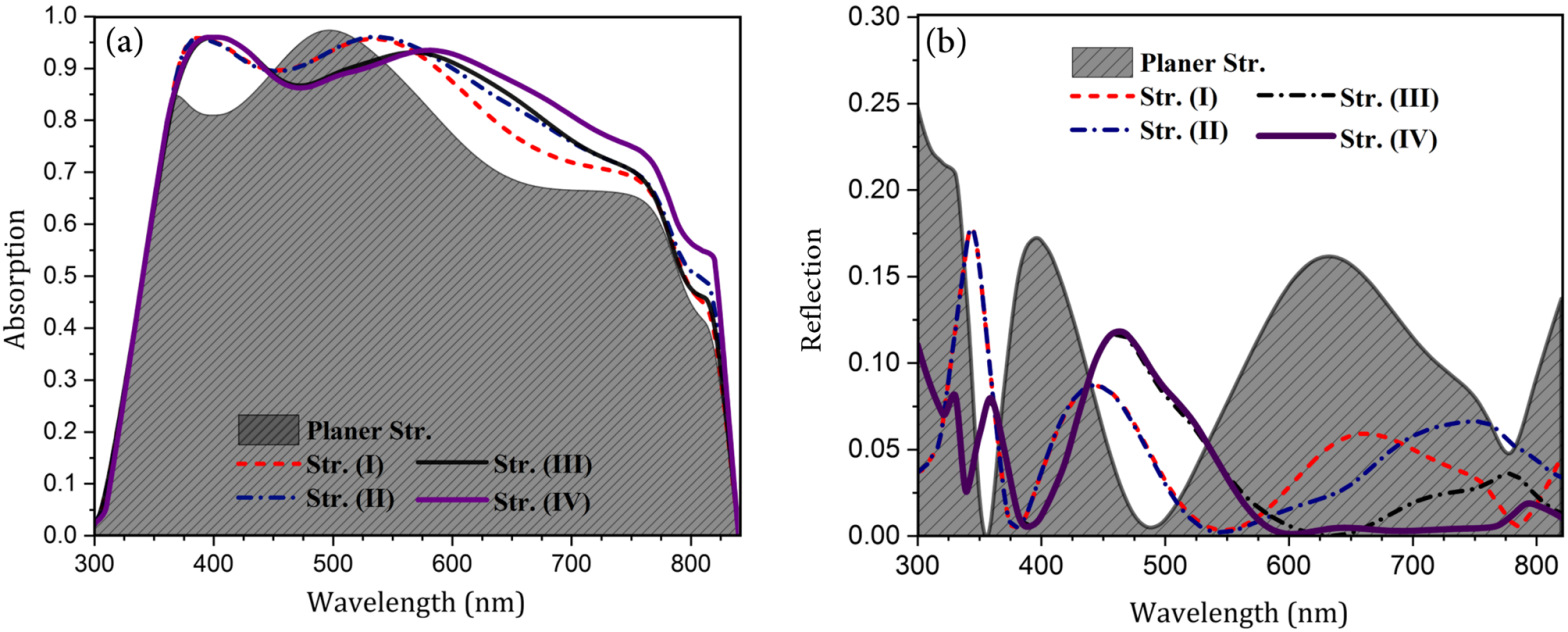
Absorption of perovskite and total reflection of the cell for planar and presented structures. (**a**) Normalized absorption of the cell. (**b**) Normalized total reflection from the cell.

### Nano-photonic structures for light absorption enhancement

Figure 3 presents the proposed photonic light-trapping structures. To accurately compare the properties of the structures, the volumes of the absorbing medium in all structures were considered equal by selecting the appropriate thickness of the perovskite. As seen in Fig. 2, in structure (I), an extra layer of Polydimethylsiloxane (PDMS) was incorporated. This layer was used as an anti-reflector due to its low refractive index and negligible absorption in the studied range of wavelength. In structure (II), the interface of perovskite and CuSCN, as well as the boundary between CuSCN and Au were taken as convex. The blue light was completely absorbed in the upper layers before reaching the HTL and did not interact with the CuSCN interface, while the red light interacted with the structure and was reflected. In structure (III), the interface of perovskite and TiO_2_, as well as the upper layers of perovskite were convex. In this structure, since the form of the light input boundary varied, the whole spectrum of incident light was affected. This nanostructure scattered the incoming light in abnormal directions and caused the light path inside the active layer to be longer. By changing the optical path inside the cell, the distribution of the electric field inside it changed completely. In structure (IV), all layers were convex, and therefore, this structure has the characteristics of all previous structures. Our target structure was the last one (i.e., structure IV), and other structures were investigated only to understand the role of nanostructures in light-trapping. To discover what happens inside the cell by changing the configuration of the structure, the absorption of perovskite and the total reflection spectrum are shown in Fig. 4. Base on this figure, in all light-trapping geometries, the amount of light absorption in the active layer increased.

Unintended Fresnel reflection on the top surface of the solar cell due to the difference of refractive indices of the two adjacent mediums reduces the light penetration into the structure and results in energy dissipation^32^. In structure (I), to reduce the reflection losses, an ARL with a lower refractive index than ITO was used at the top of the structure. In the simulation, a thin membrane of PDMS was considered as the ARL. A layer with this thickness of material could be deposited by the method presented in 2007^33^. Due to the closer refractive index of this layer to the air, the reflection from the cell surface reduced. Thus, more light entered the cell compared to the previous structure. Considering Fig. 4a,b, as expected, the total reflection decreased across almost all wavelengths of the spectrum, and this resulted in increased perovskite absorption. However, in some wavelengths, despite anti-reflective surfaces, there was an increase in reflection. These effects were caused by the destructive interference of the reflected light with the incoming light. All the structures were compared with structure (I) since the behavior of the cell has changed greatly by incorporating the ARL.

In structure (II), since nanostructures were located at the lower part of the perovskite, only longer wavelengths of the incident light were affected. This is because, as already mentioned, photons with shorter wavelengths are fully absorbed before they reach this point. It can be seen that the absorption spectrum of this structure is similar to that of the the first structure in the first half of the spectrum and all changes are seen in the second half. Due to the shape of the perovskite and CuSCN interface, the red light that reaches the boundary of these two materials collides at a non-perpendicular angle. This increases the probability of reflection into the active layer due to the closeness of the incident angle to the total reflection angle. Furthermore, this reflected light moves at a non-vertical angle inside the absorbing layer. Therefore, the optical pathlength inside the perovskite and their interaction time increase, which increases the probability of absorption. As expected, the changes in the total reflection spectrum were also seen in the second half. In the first part of these changes, there was a decrease in reflection. This represents the improvement in perovskite absorption due to the extended pathlength of light. But in the second part, there was an increase in reflection. This indicates that the reflected light from the perovskite/HTL interface was not fully absorbed in the perovskite and it left the cell.

In structure (III), the convex part was located at the top of the structure, and thus it affected the whole spectrum of the input light wave. Unlike the planar structure where the light hits the cell perpendicularly, in this structure, the ITO curvature causes the collision of the incident light to be tilted, leading to slightly increased reflection at the boundary. This convex shape of the layers changes the direction of entered light in the cell and makes it move non-vertically. Again, due to the angular light motion, its pathlength increases inside the perovskite. In addition, it reflects more at the boundaries of the absorbing layer, resulting in increased absorption. As presented in the reflection spectrum, in the second half of the spectrum, the reflection of the structure declined sharply, suggesting that the light that entered the cell hardly left it. However, at about 500 nm, the reflection increased. As mentioned, this was due to the destructive interference of the incoming wave with the reflected wave from the cell surface. This phenomenon, which reduced absorption, was mainly dependent on the thickness of the layers and not related to the nanostructure.

Structure (IV) was a combination of the two previous structures and enjoyed the properties of both. Since blue light does not interact with the underlying structure of the perovskite in this structure, at low wavelengths, the absorption of the perovskite was exactly like that in structure (III). But at wavelengths above 550 nm, the absorption in the perovskite increased compared to structures (II) and (III). This improvement is due to the properties of both structures. Overall, in this structure, in addition to reducing the light absorption in a limited portion of the spectrum due to increased reflection, the use of nanostructures greatly enhanced the perovskite absorption. As expected, the total reflection graph was similar to that of the structure (III) at short wavelengths. But, in the long wavelengths, unlike structure (III), the reflection was almost zero such that light did not leave the cell at all. This occurred because of enhanced light-trapping inside the perovskite.

**Fig. 5:**
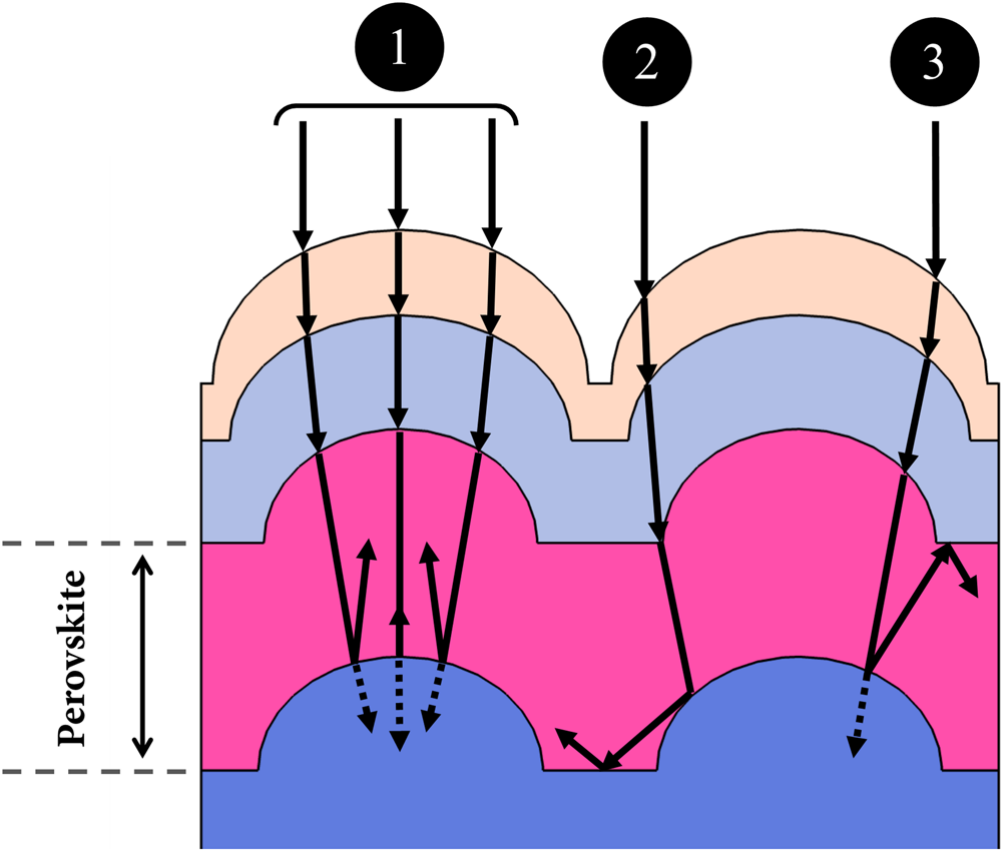
Schematic of the light-trapping principle in the convex nanostructured PSC.

**Fig. 6:**
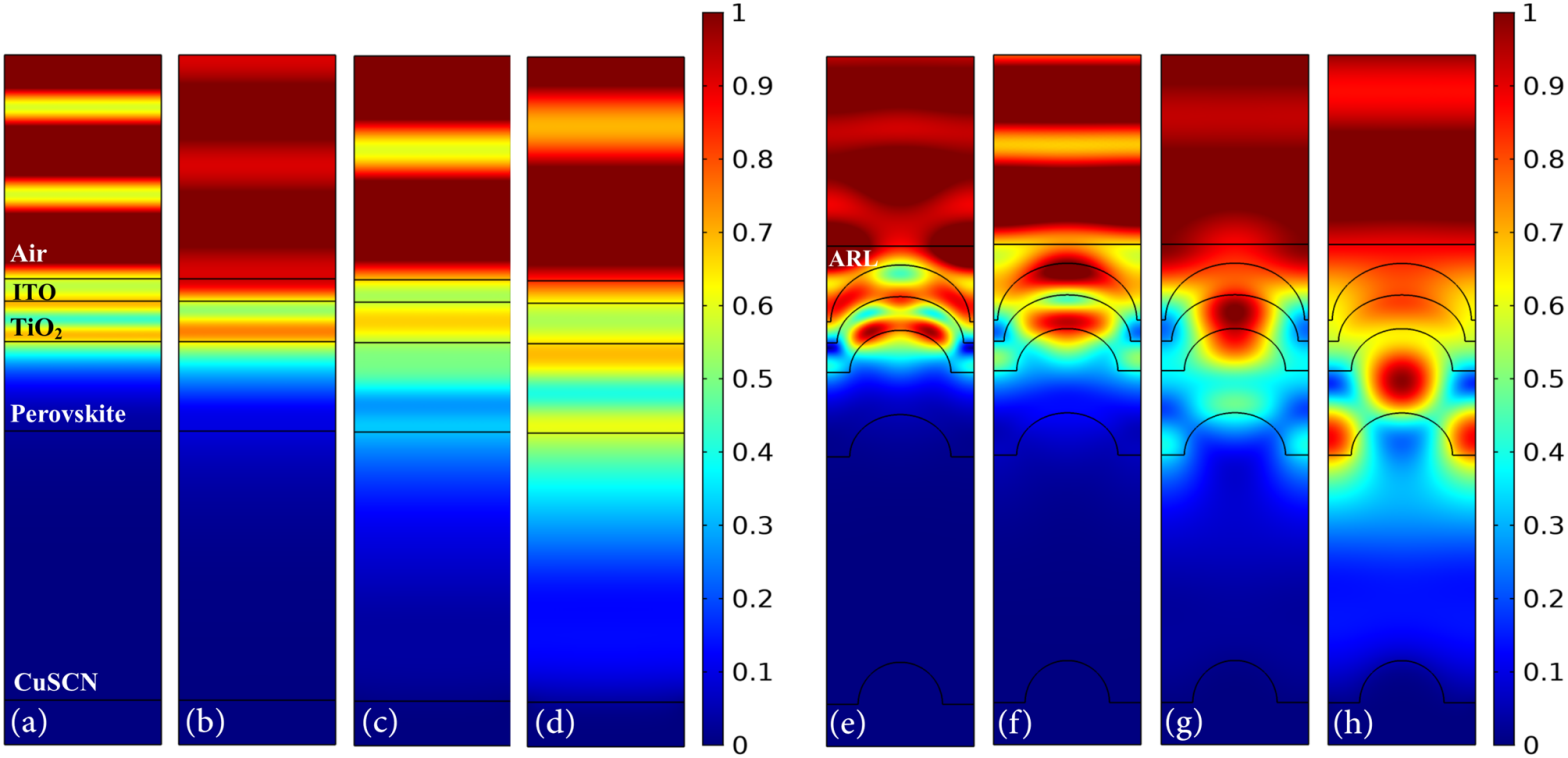
Profile of normalized electric field for the planar and proposed structures. (**a**,**e**) At the wavelength of 390 nm, (**b**,**f**) at 480 nm, (**c**,**g**) at 630 nm, and (**d**,**h**) at 800 nm.

#### Light-trapping principle and optical properties of the convex nanostructured PSC

To better understand the feature of light-trapping in structure (IV), the sketch of the light path within the cell is shown in Fig. 5. As discussed earlier, the structural shape of the layers changes the direction of moving light inside the cell. In the upper layers of the perovskite, each layer has a higher refractive index than its top layer. As a result, after the impact, light converges to the center of the cell due to angled collision. Three situations may occur for the light that reaches the perovskite/HTL interface. If it is closer to the center of the cell (condition 1), the reflected light has a path almost the same as that of the incoming light in the opposite direction. Therefore, it moves toward the ETL and another reflection occurs the same way; i.e., light is trapped in the upper region of the convex nanostructure in the perovskite. If the light that hits the interface of perovskite and HTL is more at a greater distance from the center of the cell, the reflection angle increases, and light reflects to the sides of the nanostructure. If it is too far from the center (condition 2), due to approach of the the incident angle to the total reflection angle, the probability of reflection from the boundary increases sharply. As a result, more light returns to the absorbing layer. This light hits the HTL interface again and its reflection would be much more because of the high-angle collision. But if it is not too far from the center (condition 3), the reflection angle would not be so much and it reflects toward the ETL and returns to the active layer again. Hence, in both conditions, light is trapped between the two sides of the convex nanostructure in the active layer.

Figure 6 shows the profile of the electric field for the planar structure and structure (IV) at wavelengths of 390, 480, 630 and 800 nm. As shown in Fig. 4a, the first peak in the absorption spectrum of the proposed structure appears at 390 nm. This is due to the decrease in the light reflection and the ease of its entry into the cell due to the presence of ARL. Therefore, the electric field intensity inside the cell is increased, indicating that more light entered into the cell. Furthermore, due to the shape of the nanostructure, the electric field intensity in the upper region of the perovskite increased and resulted in the enhanced absorption (Fig. 6a,e).

At a specific interval of the spectrum, the optical absorption of the planar structure is greater than the proposed structure. At 480 nm, the difference between the absorptions of these two structures reaches a maximum. At this wavelength, the intensity of the electric field outside the nanostructured cell is higher than that of the planar structure, indicating increased reflection due to the destructive interference of the incoming and reflected waves. In addition, the light that entered the nanostructured cell is trapped in the upper layers of the perovskite, especially TiO_2_, and could not penetrate the perovskite. Therefore, in this wavelength range, the proposed structure has destructive effects (Fig. 6b,f).

In the second half of the absorption spectrum, the active layer of structure (IV) generally has more absorption than the planar structure. The difference in their absorption is maximum at 630 nm. According to the profile of the electric field, as expected, the presence of ARL and convex nanostructure caused more light to enter the cell and converge to the center of the perovskite layer. As a result, more light concentrated in the absorbing layer than to the planar structure. The electric field intensity also decreased in the CuSCN layer, indicating that light was trapped in the absorbing layer and only a small amount entered the lower layer. As a result, the perovskite absorption at this wavelength increased (Fig. 6c,g).

As mentioned before, at short wavelengths, light waves cannot penetrate beyond the active layer due to the absorption of their high energy photons. Therefore, at these wavelengths, the electric field intensity is higher in the upper regions of the perovskite. But the longer the wavelength, the less is the energy of the photons and light moves longer before being absorbed. As a result, at higher wavelengths, more light reaches the bottom of the absorbing layer and scatters in abnormal directions, leading to a larger amount of light-trapping inside the perovskite. Thus, at 800 nm, compared to shorter wavelengths, the distribution of the electric field within the cell is more in line with the discussed light-trapping areas. As shown in the profile of the electric field, at this wavelength, light is trapped between the two sides of the convex nanostructure and the area above it in the active layer and entered to a smaller extent to HTL, which is consistent with the observation of the geometrical light path. This light-trapping principle of the proposed structure increased the absorption of the active layer dramatically (Fig. 6d,h).

### Plasmonic structure for light absorption enhancement

Generally, due to the higher refractive index of perovskite than other materials, the absorbing layer acts somewhat like a Fabry-Pérot cavity with partial mirrors formed by Fresnel reflection at its interfaces^20^, leading to resonance and enhancement of optical absorption. To further increase the absorption of the active layer, we used a nanostructure to divert the incident light inside the cell and make it more possible to reflect at interfaces and trap it in the active layer. But even in this case, part of the light enters HTL and is parasitically absorbed and wasted. So, a plasmonic reflector could be inserted under the active layer to increase reflection at the perovskite/HTL interface and reduce light absorption in CuSCN. A simple but efficient way is to reduce the thickness of the CuSCN layer and close the back-contact to the boundary of the absorbing layer. This reduces the parasitic absorption in the HTL and allows more light to reach the back-contact. The light that reaches the Au surface, due to the SPR effect at the metal and non-metal interface, returns to the absorbing layer, leading to a further reduction in parasitic losses and increased absorption. Considering the dimensions of the plasmonic nanostructure, the mechanism of the SPR that occurs in this structure is expected to be a far-field scattering effect^34^, which will be discussed in detail in the following section on electric field profiles.

**Fig. 7:**
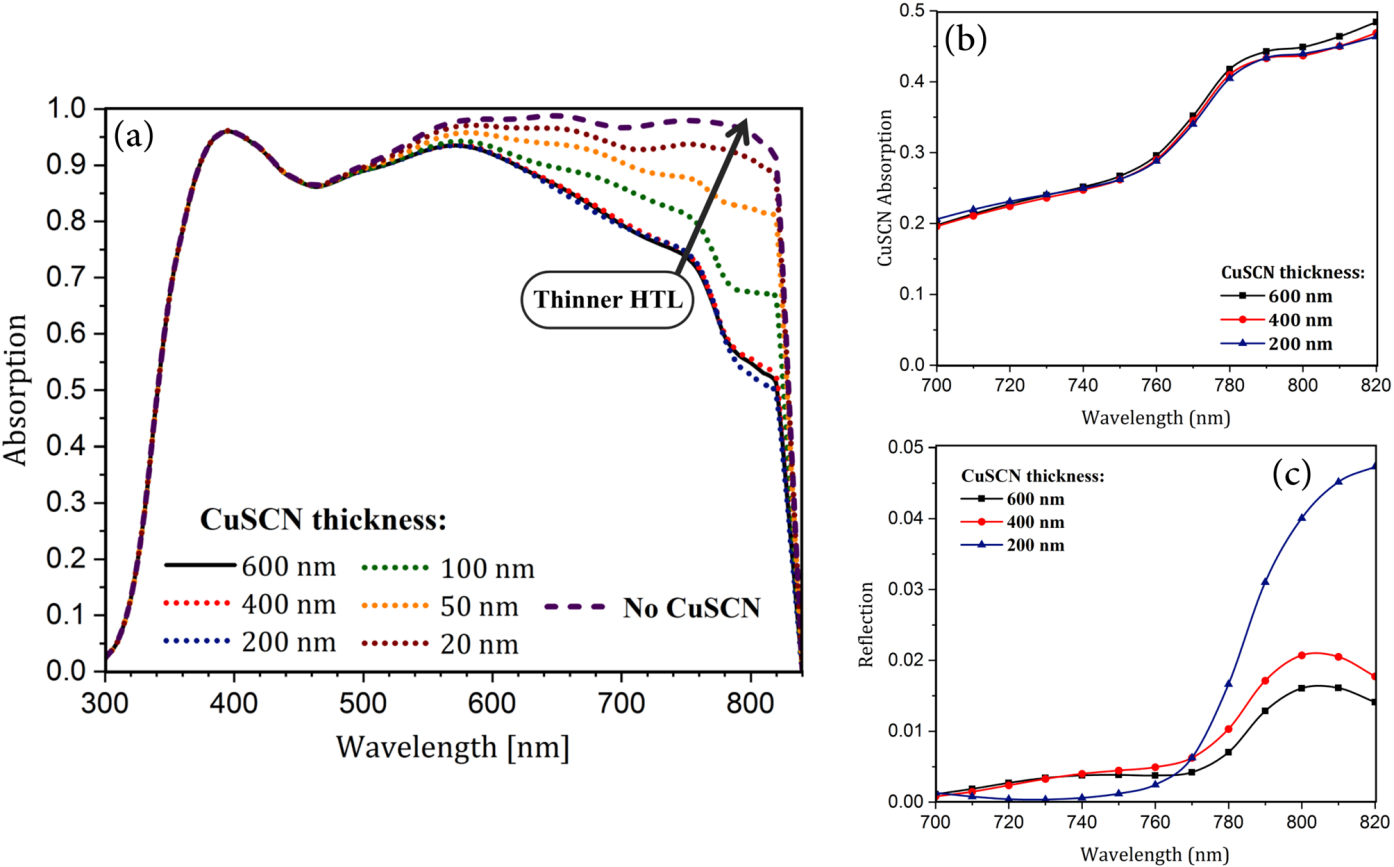
Normalized absorption and reflection of the convex nanostructured PSC (structure (IV)) with different thicknesses of HTL. (**a**) Normalized perovskite absorption of Str (IV) with the HTL thicknesses of 600, 400, 200, 100, 50, and 20 nm. (**b**) Normalized CuSCN absorption, and (**c**) normalized reflection from Str (IV) with the HTL thicknesses of 600, 400, and 200 nm.

**Fig. 8:**
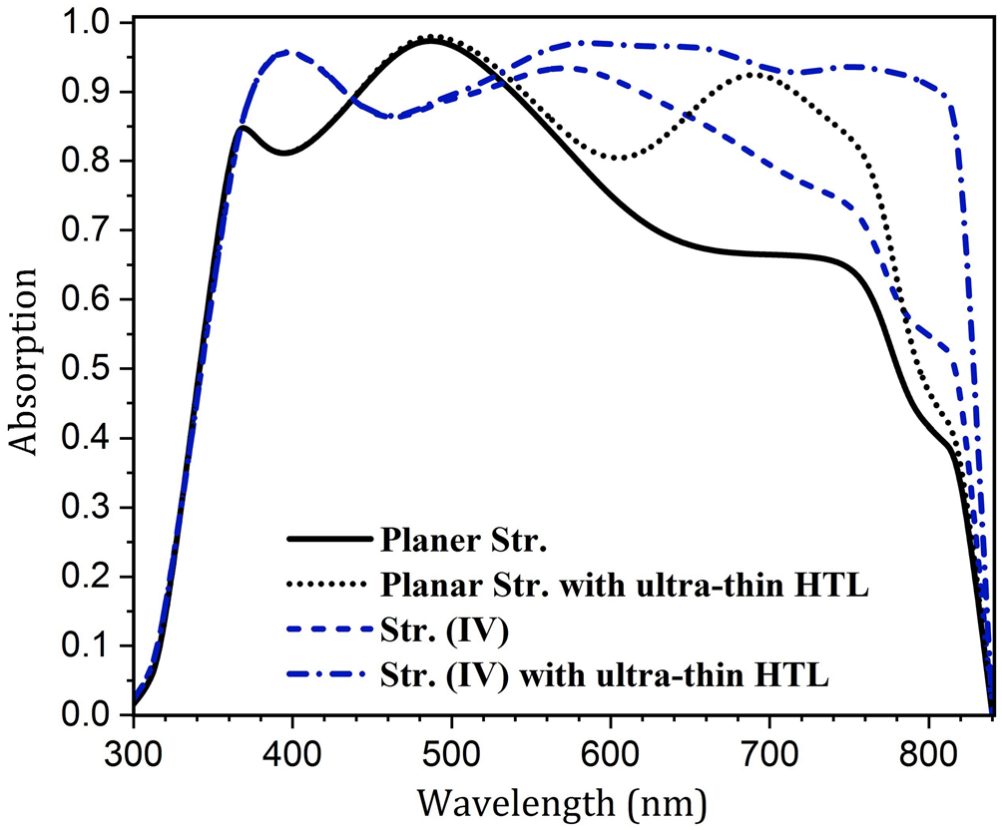
Normalized absorption of the active layer in the ultra-thin HTL structure in the presence and absence of nanostructures.

Reducing the thickness of the hole transport layer in the cell has two advantages: (1) reduction of the used expensive material in the fabrication process, and (2) improving absorption by using the SPR effect. As shown in Fig. 7a, regardless of the trend of the absorption curve and the light behavior inside the cell, decreasing the thickness of the CuSCN generally increases absorption by perovskite. It can be seen that as the thickness of HTL decreases, the parasitic losses of this layer, which otherwise causes complete light loss, decreases, and as a result, light reaches the metallic back-contact surface. This light due to the SPR effect on the metal and semiconductor interface scatters and returns to the absorbing layer. As seen in Fig. 7a, the effect of reducing HTL density on the perovskite absorption at thicknesses less than 100 nm was higher. This is because, at thicker HTLs (200 and 400 nm), the optical loss in this layer was still high, and this, to a large extent, prevented the return of scattered light to the active layer. However, contrary to expectations, the absorption of perovskite in the wavelength range of 760 to 820 nm in a cell with 200 nm HTL was slightly less than that of the higher thickness of this layer (400 and 600 nm). As shown in Fig. 2, at these wavelengths, optical losses only include parasitic absorption in CuSCN and cell reflection. Since the parasitic losses of this layer with decreasing HTL thickness decreases (Fig. 7b), the reduction in perovskite absorption is mainly due to the escape of light from the cell, and increased reflection (Fig. 7c). This is because, due to the high wavelength of light and the specific dimensions of the nanostructure, unlike other cells with thicker HTLs, scattered light from the Au back-contact (albeit very small) is not trapped in the active layer and leaves the cell. As a result, reflection increases, and the absorption of perovskite decreases.

As presented in Fig. 7a, the maximum absorption happened when there is no HTM, because in this case, maximum light intensity reaches the metallic back-contact and returns to the active layer by scattering effect. Here, for greater enhancement of plasmonic reflection, we can remove HTM. However, this, despite benefits, suffers from two drawbacks. First, with the proximity of perovskite to the metal, the stability of perovskite deteriorates and its performance degrades^35^. Secondly, recombination at the interface arises dramatically^36^. Therefore, the thickness of the CuSCN layer was taken as 20 nm. Under this condition, the absorption increases due to the plasmonic reflection and no material deterioration and increased recombination at the interface occurs. This thin layer of CuSCN can be deposited using an appropriate solvent^37^.

Since both photonic and plasmonic structures increase absorption, the scattering effect of SPR and convex light-trapping nanostructures on cell function should be investigated separately. To do this, the effect of reducing the HTL thickness was examined for both planar and the proposed (IV) structures. Figure 8 shows the absorption of the perovskite uptake in the presence and absence of convex nanostructures of a PSC with reduced thickness of CuSCN. In both structures, the absorption of the active layer increased dramatically due to the scattering of light by metal contact. This is because, in the scattering process, light couples to the SPR modes of the metal and then reradiates into the absorbing layer. In the radiation process, the electric field of these resonance modes acts as a secondary source of waves that propagate from the bottom of the perovskite. As a result, absorption increased in the second half of the spectrum. At longer wavelengths, as discussed earlier, due to the low energy of photons, light is not fully absorbed in the upper layers, and it interacts with the metal surface.

According to Fig. 8, up until 745 nm, the absorption in the planar structure increased further and at higher wavelengths, structure (IV) had better absorption. In addition, in the absorption spectrum of the planar structure, a peak occurred at 680 nm. At this wavelength, the incoming wave overlapped with a reflected wave from the back-contact and amplified the electric field inside the absorbing layer. This indicates that the light is coupled to the local modes of the active layer, resulting in a sharp increase in absorption. The wavelength of this peak depends on the thickness of the layers. Therefore, with a change in the dimensions of the layers, this wavelength changes as well. In structure (IV) with ultra-thin HTL, unlike the planar structure, there was no notable peak in the absorption spectrum of the active layer. This is because, in this structure, the reflected wave does not move parallel to the incoming one and therefore does not have destructive or constructive interference with this wave. But, due to the specific geometry of this nanostructure, light scatters in non-vertical directions, which results in coupling it to the waveguide modes of the active layer. Unlike the local modes, the waveguide modes propagate in the structure and are not confined to an area. As a result, in this condition, light is trapped in the perovskite, until it is fully absorbed.

**Fig. 9:**
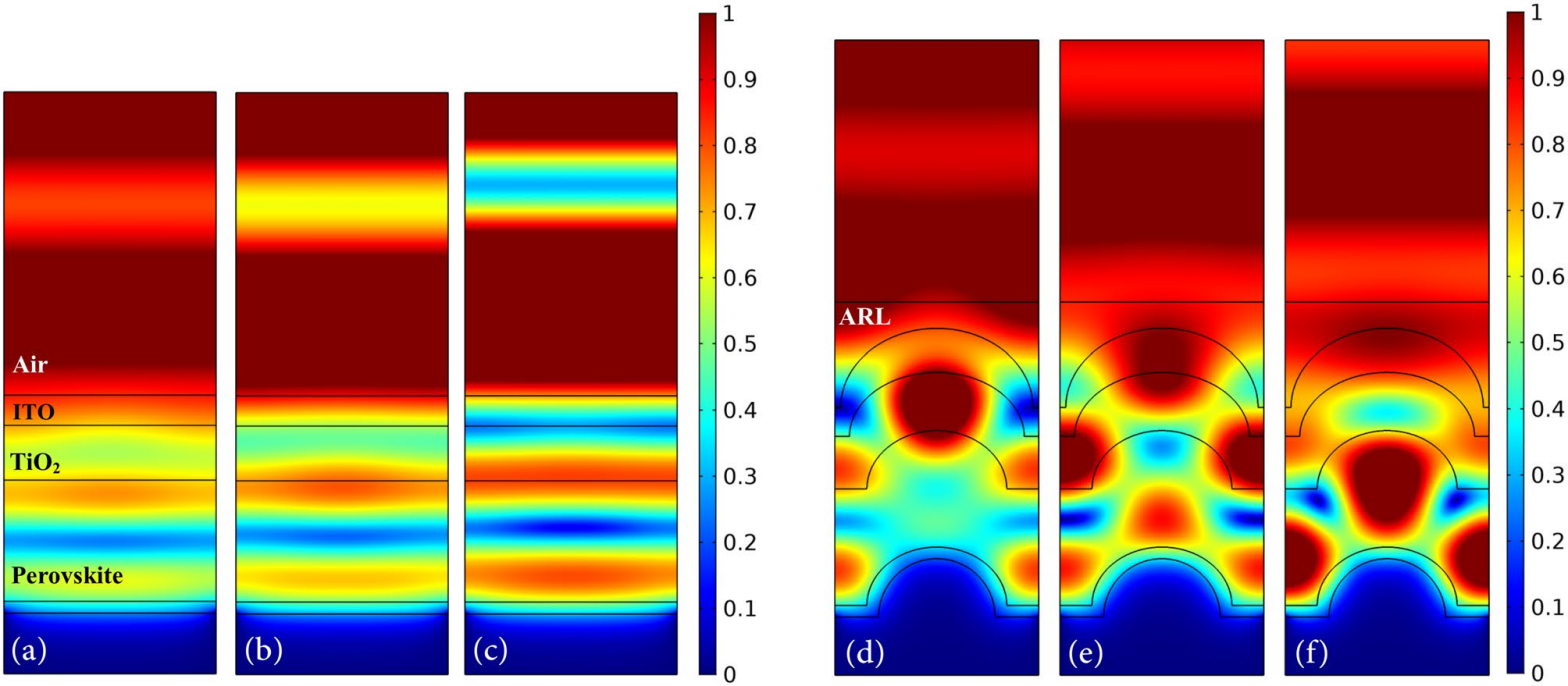
Profile of normalized electric field in ultra-thin HTL structure in the absence and presence of nanostructures. (**a**,**d**) At the wavelength of 690 nm, (**b**,**e**) at 760 nm, and (**c**,**f**) at 800 nm.

**Fig. 10:**
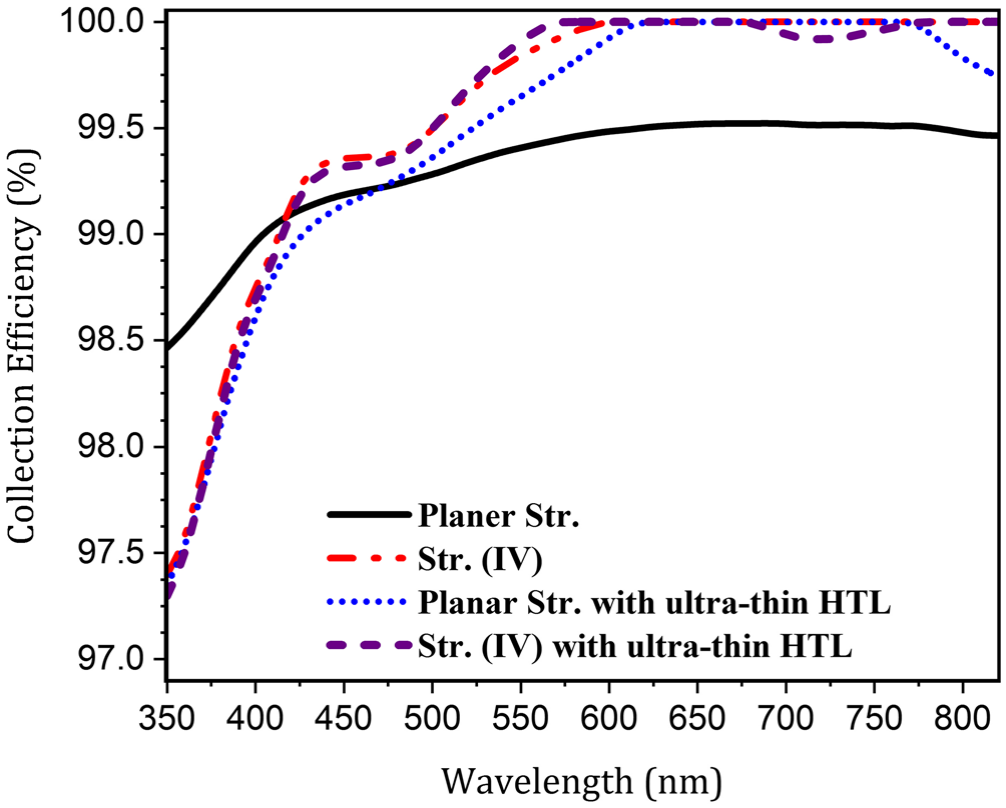
The collection efficiency spectrum of the planar and proposed nanostrcutures of PSC.

**Fig. 11:**
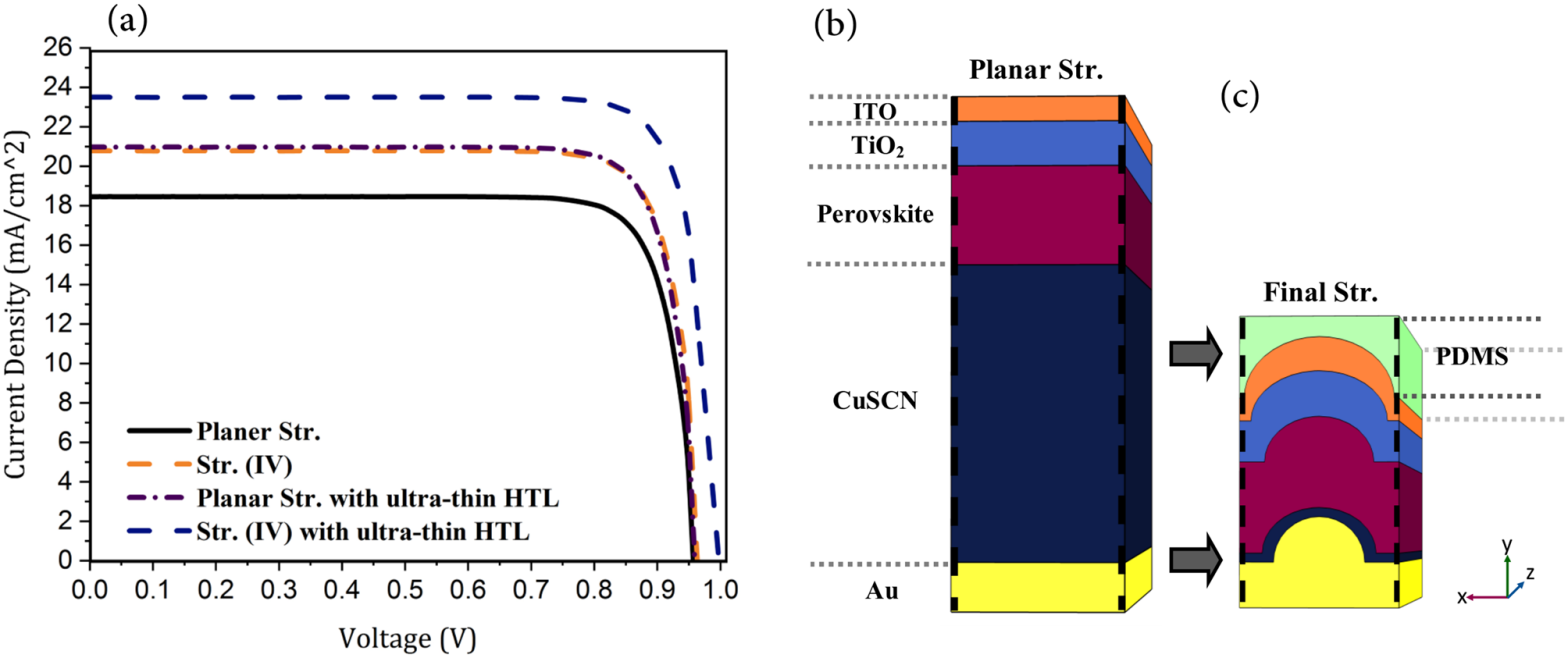
(**a**) The current–voltage (J–V) characteristics of different structures and the final model of the proposed nanostructured PSC. (**a**) The J–V characteristics of planar and proposed structures of PSC. (**b**) The planar structure of a PSC. (**c**) The final model of the proposed nanostructured PSC (structure (IV) with ultra-thin HTL).

Figure 9 shows the electric field profile of the planar structure and convex nanostructure of a PSC with ultra-thin HTL. The comparison of the electric field profiles of the planar structures with conventional and ultra-thin HTL (Figs. 6 and 9) shows that there was no notable change in the distribution of the electric field. Only the intensity of the electric field within the cell increased due to the SPR enhancement. This indicates that the back-contact only acts as a reflector that scatters the light towards the perovskite and does not confine it in the vicinity of the planar metallic contact. It can be said that the mechanism of SPR in this structure is far-field scattering and there are no near-field effects. However, considering the electric field at the metal surface, a small amount of light is absorbed by the Au contact. But this is insignificant compared to scattered light. Furthermore, since the absorption peak for the planar structure was at 690 nm, one can see most of the electric field intensity within the absorbing layer was at this wavelength.

The comparison between the electric field profiles of the two convex nanostructures with conventional and ultra-thin HTLs (Figs. 6 and 9) shows that the distribution of the electric field in these two structures is also similar and, like the planar structure, only the intensity of the electric field increased in the plasmonic nanostructure. As presented in the profile of the electric field, there is no local confinement of the electric field in the vicinity of the plasmonic nanostructure, indicating that there are no near-field effects of SPR in this configuration. This is because, the size of the convex nanostructure is large (> 100 nm) and the increase in the absorption is dominantly due to the far-field scattering effects rather than near-field enhancement. Therefore, similar to the planar structure, the metallic back-contact acts as a scatterer and reflects light to the active layer. But since the scattered light from the convex nanostructure is directed toward the sides of the cell, in this structure unlike in the planar, it couples to the guided modes of the absorbing layer. These modes do not leave the cell and propagate inside it until their energy is absorbed. As a result, in the wavelengths at which SPR occurs, absorption enhances dramatically. At 800 nm, the high intensity of the electric field in the absorbing layer indicates that light is trapped entirely in this layer due to the coupling to the waveguide modes. Therefore, at this wavelength, it had the highest absorption. But at 760 nm, light is not coupled fully to the waveguide modes of the active layer. So the absorption at this wavelength was slightly lower than that at 800 nm.

### Electrical study of proposed photonic and plasmonic structures

All analyses, including the absorption of structure and the distribution of the electric field inside the cell, were hitherto in terms of optical properties. In this section, to investigate the electrical properties of the PSCs, the current–voltage (J–V) characteristic and the collection efficiency spectrum of the cells are discussed. This spectrum is obtained based on a relationship similar to that of the internal quantum efficiency relation with some subtle differences. The collection efficiency shows the proportion of carriers extracted by contacts to all the generated carriers inside the active layer. This ratio indicates the extent to which photo-generated carriers in the active layer resulted in electrical current at the corresponding wavelength.

As shown in Fig. 10, in the presented structures, the collection efficiency improved at wavelengths above 430 nm. This is because, in these structures, due to the use of ARL, the penetration of light into the cell is high. As a result, the distribution of the electric field in the cell becomes more uniform, causing the generated carriers to be closer to the HTL interface. Therefore, due to improved carrier extraction, the collection rate reaches a maximum. In convex nanostructures, due to the curvature of the HTL and perovskite boundary, the carrier extraction medium is closer to the generation site of carriers. Therefore, the collection rate in these structures is even better than the planar structure.

However, according to Fig. 10, the collection efficiency deteriorated at wavelengths shorter than 430 nm. This can be attributed to the fact that at low wavelengths, light is absorbed mostly in the upper part of the perovskite. Therefore, considering the electric field profiles (Figs. 6 and 9), at wavelengths lower than 400 nm, due to the specific configuration of the nanostructured PSC, the carriers are generated in a small area of the upper convex region of the active layer. Accordingly, compared to the planar structure, they have a lower interface with the ETL. As a result, they are harder to collect, leading to a decrease in the collection rate in this range. On the other hand, as presented in Fig. 4a, the absorption of light in these wavelengths was generally low and did not have much effect on the output current of the cell. So, overall, in the presented nanostructure with an ultra-thin HTL, there is better carrier collection than in the conventional planar structure of PSC.

Reduction of recombination at contacts and improving the carrier’s transmission in the cell decrease the hysteresis behavior of PSC^38^. Therefore, hysteresis is expected to decrease in the plasmonic nanostructure due to improvement in carrier extraction and reduction of charge recombination by enhancement of collection efficiency (see Fig. 10)^39,40^. However, the behavior of hysteresis should be investigated experimentally using actual cells because the quality of the electrical contacts of the layers and interfacial recombination resulting from it (such as recombination through defect levels) depend on the nature of the manufacturing process^41^.

As shown in Fig. 11a, in the presented photonic and plasmonic structures, the number of photo-generated carriers in the cell and consequently the current density of the cell increased due to increased light absorption. With the increase of the current in these structures, the PCE of the cells improved as well. But since the change in absorption only causes a change in the output current and its corresponding PCE and does not have a significant impact on other parameters, the change in V_oc_ is not dependent on the absorption of the system and is due only to the increased carrier extraction and improved collection efficiency. Furthermore, FF showed almost no change, since the used materials had not changed.

**Table 1:** The short circuit current density (J_sc_), the open circuit voltage (V_oc_), the filling factor (FF), and the conversion efficiency of the PSCs with different structures.

Structure	Planar	(I)	(II)	(III)	(IV)	Plasmonic planar	Plasmonic (IV)
${J} _{{sc}}$ (mA/cm^2^)	18.63	19.67	20.26	20.03	20.78	20.97	23.50
${V} _{{oc}}$ (V)	0.9564	0.9568	0.9595	0.9606	0.9625	0.9585	0.9979
FF (%)	82.84	82.94	82.94	83.24	83.45	83.11	83.31
PCE (%)	14.62	15.60	16.18	16.02	16.69	16.70	19.54

**Table 2:** The electrical parameters of the simulated PSC.

Parameter	${TiO} _{{2}}$	Perovskite	CuSCN
${{\varepsilon }}_{ {r}}$	9	6.5	10
${N}_{ {C}}$ (${\text{cm}}^{-3}$)	$1\times 10^{19}$	$5.41\times 10^{19}$	$2.51\times 10^{19}$
${N}_{ {V}}$ (${\text{cm}}^{-3}$)	$1\times 10^{19}$	$1.66\times 10^{19}$	$1.79\times 10^{19}$
${{\mu }}_{ {n}}/{{\mu }}_{ {p}}$ (${\text{cm}}^2/V.s$)	20/10	50/50	$1\times 10^{-4}/0.01$
${ {X}}$ (*eV*)	4	3.93	1.9
Eg (*eV*)	3.2	1.5	3.4
Doping (${\text{cm}}^{-3}$)	$5\times 10^{18}$	$5\times 10^{13}$	$5\times 10^{18}$
${{\tau }}_{{n}}/{{\tau }}_{{p}}$ (ns)	5/2	8/8	5/5

According to Table 1, the J_sc_ in structure (IV) had a value of 20.78 mA/cm^2^, the highest compared to those of planar structure and structures (I), (II), and (III), which is consistent with the absorption analyses of these structures. In addition, on using the plasmonic reflector in the planar structure, the current density increased so that J_sc_ reached a value of 20.97 mA/cm^2^, indicating that the increase of J_sc_ in the plasmonic structure is slightly greater than that of the convex nanostructure. But the structure (IV) with ultra-thin HTL (Fig. 11c), which is the integration of plasmonic and nano-photonic structure, had the highest J_sc_ and V_oc_ of all. This is due to the combination of the properties of the photonic and plasmonic structures. In this structure, J_sc_ and V_oc_ were 23.5 mA/cm^2^ and 0.99 V, respectively, and PCE was 19.54%, which was 33% better than the conventional planar structure (Fig. 11b).

## Discussion

In the present study, nanostructured PSC with a constant thickness of the absorbing layer investigated in order to reduce the optical losses of the conventional planar structure and increase the efficiency of the cell. First, using an anti-reflection layer on the top of the cell, the reflection from the cell surface was reduced. Then, convex nanostructures were used to trap light inside the cell and increase its absorption. The manner in which these nanostructures in each layer enhanced absorption by reflecting light at the perovskite boundary and increasing its pathway within the cell, was demonstrated. The impact of this nanostructure on the improved extraction of the generated carrier considering the distribution of the electric field inside it was investigated. The results showed that using this structure, J_sc_ and PCE increased by 11% and 14%, respectively. Furthermore, a plasmonic scatterer was used beneath the perovskite to further enhance the cell efficiency. The mechanism of the SPR according to the distribution of the electric field inside the cell was investigated. The increase in the absorption through scattering of light in the structure was shown. By inserting the plasmonic scatterer in planar and photonic structures, the SPR effects on both structures were investigated separately. In the final geometry, which was a combination of the light-trapping structure and the plasmonic scatterer, the absorption was shown to increase sharply resulting in PCE value of 19.5%, which is 33% higher than that of the planar PSC.

## Methods

A coupled optical and electrical model is used for the investigation of the nanostructured PSCs. The optical model is based on the interaction between the electromagnetic waves with the solar cell device. Maxwell’s equations can be used to describe the physics of propagation electromagnetic waves of light. Thus, in the modeling of the optical properties, the Helmholtz equations, derived from the Maxwell equations in the frequency domain, were calculated. The input light power was based on AM1.5 spectrum and simulation was performed in the wavelength range of 300 to 820 nm with a resolution of 10 nm. The reason for choice of this wavelength range is that the highest solar light energy on earth starts from 300 nm. Furthermore, the bandgap of the absorbing layer is 1.5 eV, which makes it incapable of absorbing light at wavelengths above 820 nm. The complex refractive index of Au, CuSCN, Perovskite, TiO_2_, and ITO, as a function of $\lambda$, was obtained from previous studies^27,42–45^. After calculating the electric field in the structure, the photo-generation rate at each wavelength was obtained using the following equation:

1 $$\begin{aligned} G(\lambda )=\frac{\varepsilon ^{\prime \prime } |E|^2}{2h} \end{aligned}$$

where E is electric field, h is plank constant, and $\varepsilon ^{\prime \prime }$ is the imaginary part of the relative permittivity, which is a function of the wavelength of propagating light. Base on this relation, the number of photo generated carriers is proportional to the intensity of the electric field and the imaginary part of the permittivity, which is itself related to the refractive index and the extinction coefficient of the materials. The absorption spectrum of the active layer and the reflection spectrum of the solar cell were also obtained using the S parameters. The values obtained from this relation are independent of the input light power and only show the percentage of the absorbed or reflected light at each wavelength.

In the electrical part simulation of the photovoltaic solar cell, the current–voltage (J–V) characteristics of a solar cell is defined as:

2 $$\begin{aligned} J(V)=J_{dark}+J_{sc}=J_{0}\left( \left( exp\frac{eV}{nKT}\right) -1\right) -J_{sc}(G_{Photo}) \end{aligned}$$

The term J_dark_ represents the current of the cell when there is no illumination. This parameter is a function of the applied voltage (V) to the cell and does not depend on the photo-generated carriers. In J_dark_ calculation, J_0_ is the photovoltaic cell’s saturation current, e is the electron charge, K is the Boltzmann’s constant, T is the temperature in degrees kelvin, and n is an ideality factor which is dependent on the material’s type. In contrast, the term J_sc_ represents the solar cell current when it irradiated and no voltage applied.

Continuity and Poisson Equations were used to calculate both J_sc_ and J_dark_. However, J_sc_ was calculated by considering the number of photo-generated carriers obtained from optical calculations. J_dark_ was calculated by setting the carrier generation values to zero. This parameter was measured only to obtain the J–V characteristics of the cell. In both cases, carrier recombination during the transport within the device was considered.

The main carrier loss factor in photovoltaic devices is band-to-band or direct recombination. Therefore, in this study, the direct recombination of carriers was calculated. In addition, the Shockley Read Hall (SRH) recombination, which is a common recombination mechanism in the PSCs^46^, was considered. The ohmic contact for Au and the Schottky contact condition for ITO were used. The numerical parameters of the electrical part simulation are presented in Table 2^31,47–57^. The simulation model in this study was the finite element method (FEM). FEM is a numerical method used to compute boundary value problems for partial differential equations.

## Data Availability

The data generated and analyzed during the study are available from the authors on reasonable request.
